# Occult Blood in Urine

**Published:** 1913-04

**Authors:** 


OCCULT BLOOD IN URINE. Leed, *Medizinische Klinic*, Berlin, filters a large quantity of urine and applies the test to the residue in the filter. Naturally, this concentrates whatever corpuscles may be present. The mixture is heated to destroy ferments which might produce a reaction similar to that of haemoglobin. (Note—We recall "losing a job" in our early experience because we would not report categorically yes or no regarding the presence of blood in urine. We had found by experiment that a few drops of blood added to 500 c.c. of urine would not give a reaction with guaiac and that the red cells could not always be found microscopically after such an addition. It should be remarked, however, regarding Leed's suggestion, which is not a novel one, that it is often desirable to know whether haemoglobin has been diffused into the filtrate or not, and that red cells can also be found by the microscope, about as readily as the guaiac test can be obtained from the residue. Heating to prevent pseudo-reactions is applicable to faeces, stomach contents, etc., although, without this precaution, a blood test is not found in the absence of blood unless a very delicate test is employed, and, for clinical purposes, a very delicate test is usually undesirable. A further caution is necessary. As is well known, the blue color with guaiac gradually developes even in the absence of blood and a general rule is to pay no attention to a reaction delayed more than a minute. In fact, if any appreciable quantity even of occult blood is present, the reaction is practically instantaneous. Heat hastens the appearance of the final reaction and false impressions may be obtained unless the investigandum, heated to kill ferments, is subsequently cooled before testing for haemoglobin. And, by the way, the phenolphthalin reaction is more convenient and is said to be more reliable than that by guaiac. Aloin, benzidene, etc., seem to have no advantage over guaiac.—Ed.)

